# Tet methylcytosine dioxygenase 2 inhibits atherosclerosis via upregulation of autophagy in ApoE^−/−^ mice

**DOI:** 10.18632/oncotarget.13121

**Published:** 2016-11-04

**Authors:** Juan Peng, Qin Yang, A-Fang Li, Rong-Qing Li, Zuo Wang, Lu-Shan Liu, Zhong Ren, Xi-Long Zheng, Xiao-Qing Tang, Guo-Hua Li, Zhi-Han Tang, Zhi-Sheng Jiang, Dang-Heng Wei

**Affiliations:** ^1^ Institute of Cardiovascular Disease, Key Lab for Arteriosclerology of Hunan Province, University of South China, Hengyang, Hunan, China; ^2^ Department of Biochemistry and Molecular Biology, The Libin Cardiovascular Institute of Alberta, The University of Calgary, Health Sciences Center, Calgary, Alberta, Canada; ^3^ Department of Physiology & Institute of Neuroscience, Medical School, University of South China, Hengyang, PRC, China

**Keywords:** TET2, autophagy, endothelial cells, inflammation, atherosclerosis, Pathology Section

## Abstract

Tet methylcytosine dioxygenase 2 (TET2) mediates the conversion of 5-methylcytosine (5mC) to 5-hydroxymethylcytosine (5hmC). The loss of TET2 is associated with advanced atherosclerotic lesions. Our previous study showed that TET2 improves endothelial cell function by enhancing endothelial cell autophagy. Accordingly, this study determined the role of TET2 in atherosclerosis and potential mechanisms. In ApoE^−/−^ mice fed high-fat diet, TET2 overexpression markedly decreased atherosclerotic lesions with uniformly increased level of 5hmC and decreased level of 5mC in genomic DNA. TET2 overexpression also promoted autophagy and downregulated inflammation factors, such as vascular cell adhesion molecule 1, intercellular adhesion molecule 1, monocyte chemotactic protein 1, and interleukin-1. Consistently, TET2 knockdown with small hairpin RNA (shRNA) in ApoE^−/−^ mice decreased 5hmC and increased 5mC levels in atherosclerotic lesions. Meanwhile, autophagy was inhibited and atherosclerotic lesions progressed with an unstable lesion phenotype characterized by large lipid core, macrophage accumulation, and upregulated inflammation factor expression. Experiments with the cultured endothelial cells revealed that oxidized low-density lipoprotein (ox-LDL) inhibited endothelial cell autophagy. TET2 shRNA strengthened impaired autophagy and autophagic flux in the ox-LDL-treated endothelial cells. TET2 overexpression reversed these effects by decreasing the methylation level of the Beclin 1 promoter, which contributed to the downregulation of inflammation factors. Overall, we identified that TET2 was downregulated during the pathogenesis of atherosclerosis. The downregulation of TET2 promotes the methylation of the Beclin 1 promoter, leading to endothelial cell autophagy, impaired autophagic flux, and inflammatory factor upregulation. Upregulation of TET2 may be a novel therapeutic strategy for treating atherosclerosis.

## INTRODUCTION

Atherosclerosis, a complex disease that involves chronic inflammation and vascular remodeling processes, is the leading cause of death and morbidity among adults in developed countries. Recently, increasing evidence has indicated that autophagy is impaired during atherosclerotic plaque development and contributes to lipid metabolism dysfunction and vascular endothelial cell (EC) dysfunction [1-3].

Autophagy is a dynamic process of recycling in which cells degrade malfunctioning or damaged proteins and organelles within their lysosomes; this process plays a crucial role in cellular homeostasis. DNA methylation of some key autophagy-related (ATG) genes regulates autophagy. LC3I, an important positive regulator of autophagy, is frequently methylated in esophageal squamous-cell carcinoma [4]. Autophagy-related protein Beclin 1 promoter displays aberrant methylation, and its expression is significantly downregulated in breast tumors [5].

Methylation of cytosine at CpG sequences of the promoter is an epigenetic modification linked to gene expression regulation. Alterations in DNA methylation profiles are early markers of atherosclerosis in ApoE^−/−^ mice, and atherogenic lipoproteins induce DNA hypermethylation in cultured cells [6]. 5-Methylcytosine (5mC) is elevated in the intima of ApoE^−/−^ mice fed a high-fat diet (HFD), and high 5mC levels are linked to low-density lipoprotein (LDL) receptor and p53 mutation in vascular cells. Inhibition of DNA methylation by 5-Aza-2′-deoxycytidine (5Aza) or DNA methyltransferase by siRNA obviously inhibits endothelial inflammation and lesion formation [7]. This finding suggests that the balance between DNA methylation and demethylation is disrupted during atherosclerosis and that DNA methylation is predisposed to the pathogenesis of atherosclerosis.

Tet methylcytosine dioxygenase 2 (TET2), a member of the Ten-eleven translocation (TET) enzyme family, converts 5mC to 5hmC and modifies DNA methylation status [8, 9]. The loss of TET2 is associated with an increased hematopoietic stem cell self-renewal and skewed differentiation favoring monocytic lineage [10-12]. TET2^−/−^ mice are prone to develop myeloid malignancies, including chronic myelomonocytic leukemia and myeloproliferative neoplasm-like myeloid leukemia. Moreover, TET2′s loss of production of 5hmC is a novel epigenetic hallmark for melanoma. Recently, Liu et al. [13] have shown that TET2 is associated with atherosclerosis *via* a master epigenetic regulator of smooth muscle cell differentiation. Our previous study showed that the inhibition of TET2 expression in oxidized-LDL (ox-LDL)-treated macrophages contributes to autophagy impairment and lipid accumulation in macrophages [14]. Meanwhile, the inhibition of TET2 expression in vascular ECs under low shear stress promotes autophagy impairment in ECs and EC dysfunction *via* the upregulation of endothelial nitric oxide synthase and the downregulation of endothelin-1 [15]. However, whether TET2 dysregulation is directly implicated in the initiation and progression of atherosclerosis remains to be determined.

In the present study, we investigated the role of TET2 in atherosclerosis and its potential mechanisms. Results showed that the levels of TET2 and its product 5hmC decreased in atherosclerotic lesions. The downregulation of TET2 increased atherosclerotic lesions with increased macrophage accumulation and inflammatory factor expression in HFD-fed ApoE^−/−^ mice. Mechanistically, TET2 promoted autophagic flux in endothelial cells by increasing Beclin 1 promoter demethylation.

## RESULTS

### TET2 and 5hmC are decreased and 5mC is increased in atherosclerotic lesions in ApoE^−/−^ mice

**Figure 1:**
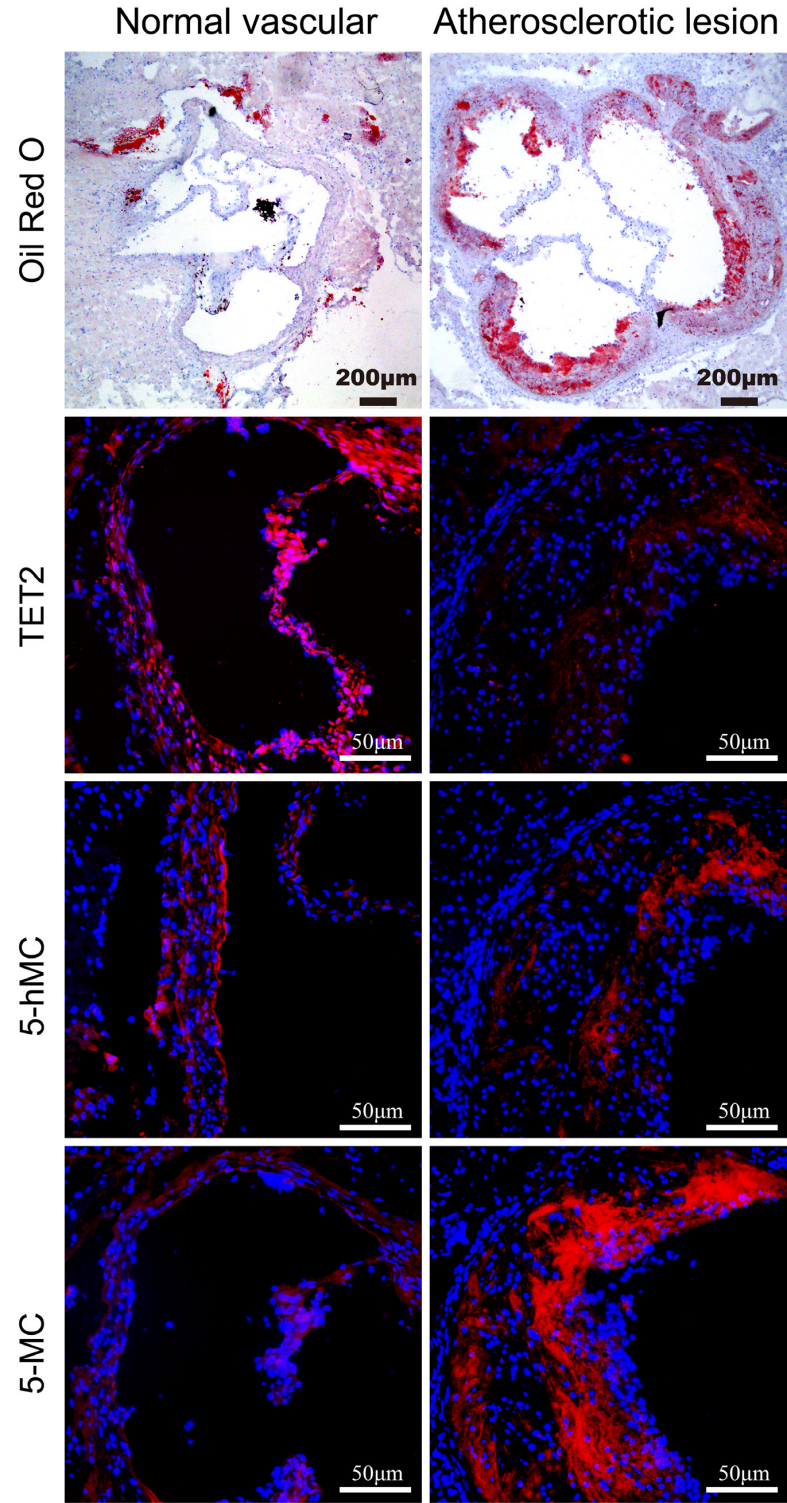
Distribution of TET2, 5hmC, and 5mC in atherosclerotic lesions and normal vascular tissue Immunostaining was performed using antibodies against TET2, 5hmC, and 5mC in normal vascular and atherosclerotic tissues in ApoE−/^−^ mice (*n* = 10 each). Cell nuclei were counterstained with DAPI. Scale bars are 200 and 50 μm.

To determine the role of TET2 in atherosclerosis, we first analyzed the extent of TET2 in normal vascular tissue and atherosclerotic lesions in ApoE^−/−^ mice. TET2 expression was homogeneously present across the vascular wall in atherosclerosis-free arteries. TET2 expression was barely detected in the intima of atherosclerotic lesions but was abundant in the shoulder regions. In addition, the production of 5hmC decreased while that of its substrate 5mC increased in atherosclerotic lesions compared with the corresponding normal vascular tissue (Figure 1). Overall, these data suggest that TET2 downregulation contributes to the formation and progression of atherosclerotic plaques.

### TET2 significantly reduces the formation of atherosclerotic lesions in ApoE^−/−^ mice

We further explored the effects of TET2 intervention (TET2 overexpression or TET2 small hairpin RNA [shRNA]) on atherosclerotic lesions in HFD-fed ApoE^−/−^ mice. We examined the levels of TET2, 5mC, and 5hmC in atherosclerotic lesions in mice from each group. As anticipated, TET2 overexpression positively correlated with 5hmC and negatively correlated with 5mC levels. Treatment with TET2 shRNA decreased 5hmC level but increased 5mC level in ApoE^−/−^ mice (Supplementary Figure 1).

**Figure 2:**
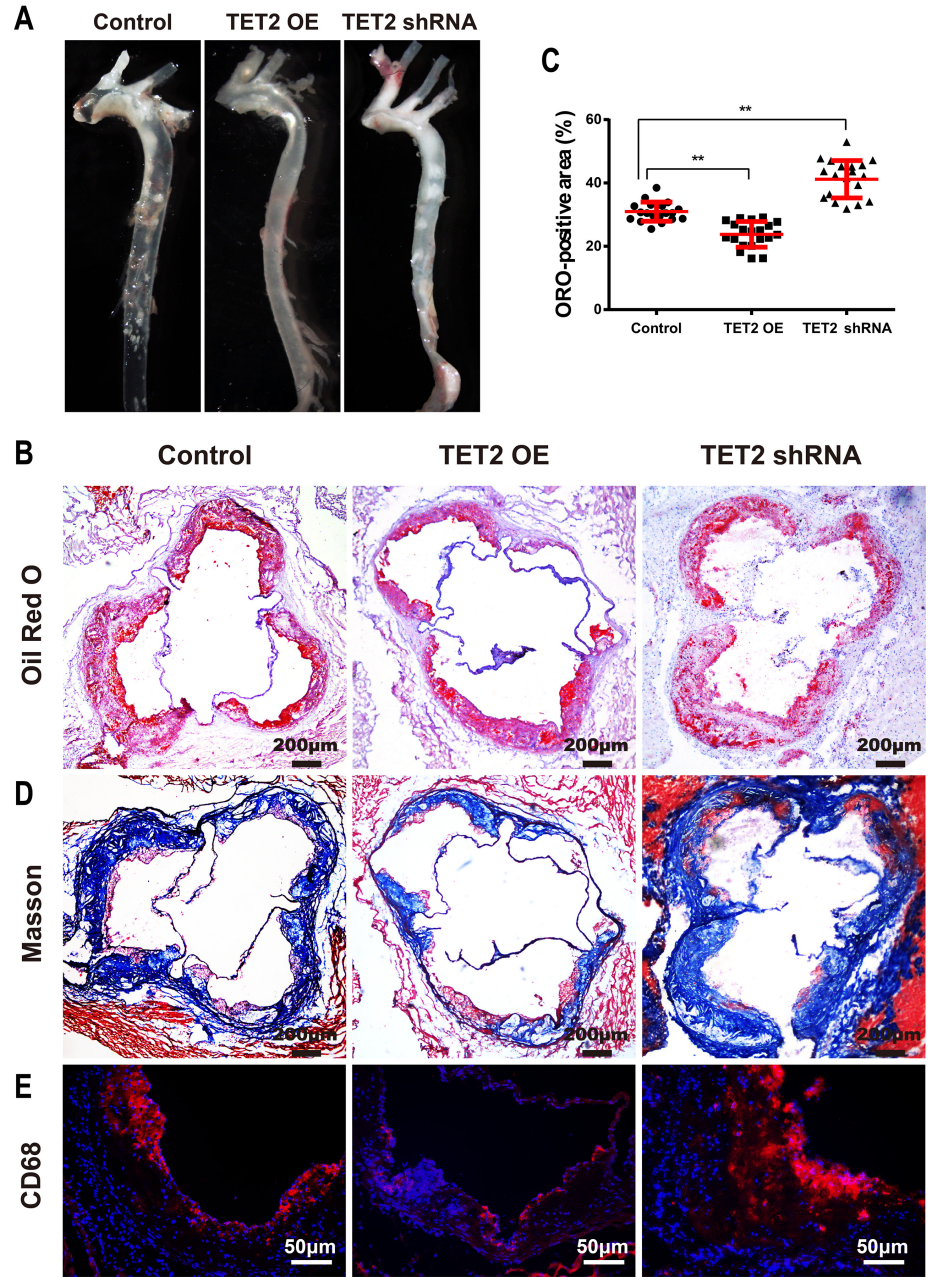
TET2 inhibited the atherosclerotic lesion progression of high fat-fed ApoE^−/−^ mice **A.** Representative en face preparations of the total aorta in ApoE−/− mice treated with TET2 overexpression or TET2 shRNA lentiviruses fed a high-fat diet for 12 weeks. **B.**-**C.** Representative microscopic images and quantification of atherosclerotic plaque areas in cross-sections of aortic roots with Oil-red staining in ApoE−/^−^ mice treated with TET2 overexpression or TET2 shRNA lentiviruses fed a high-fat diet for 12 weeks. Values are mean ± SEM (*n* = 20 each). **D.**-**E.** Representative microscopic images of the aortic sinus with Masson's Trichrome staining and immunostaining for CD68 (a macrophage marker) in ApoE−/− mice treated with TET2 overexpression or TET2 shRNA lentiviruses fed a high-fat diet for 12 weeks. **P < 0.01 compared with the control group. TET2 OE:TET2 overexpression.

No difference in body weight, total cholesterol, triglyceride, HDL and LDL levels was found among the mice in the three groups (Supplementary Figure 2). En face preparations of the total aorta (Figure 2A) showed less plaque in the TET2 overexpression group and more plaque in the TET2 shRNA group compared with the control mice. The plaque area in the proximal aorta was extensively examined in the Oil-red-stained cross-sections of the aortic root. We observed that TET2 overexpression significantly decreased atherosclerotic lesions, but treatment with TET2 shRNA significantly increased the lesions in the aortic root compared with those in control mice (Figures 2B and 2C). A more random orientation and less organized structure of lesional collagen fibrils were also observed in the TET2 shRNA-treated group than in the control group (Figure 2D). Meanwhile, the accumulation of CD68-positive macrophages decreased in the TET2 overexpression group but significantly increased in the TET2 shRNA-treated group (Figure 2E).

### TET2 reduces the expression of pro-inflammatory cytokines in plaques

**Figure 3:**
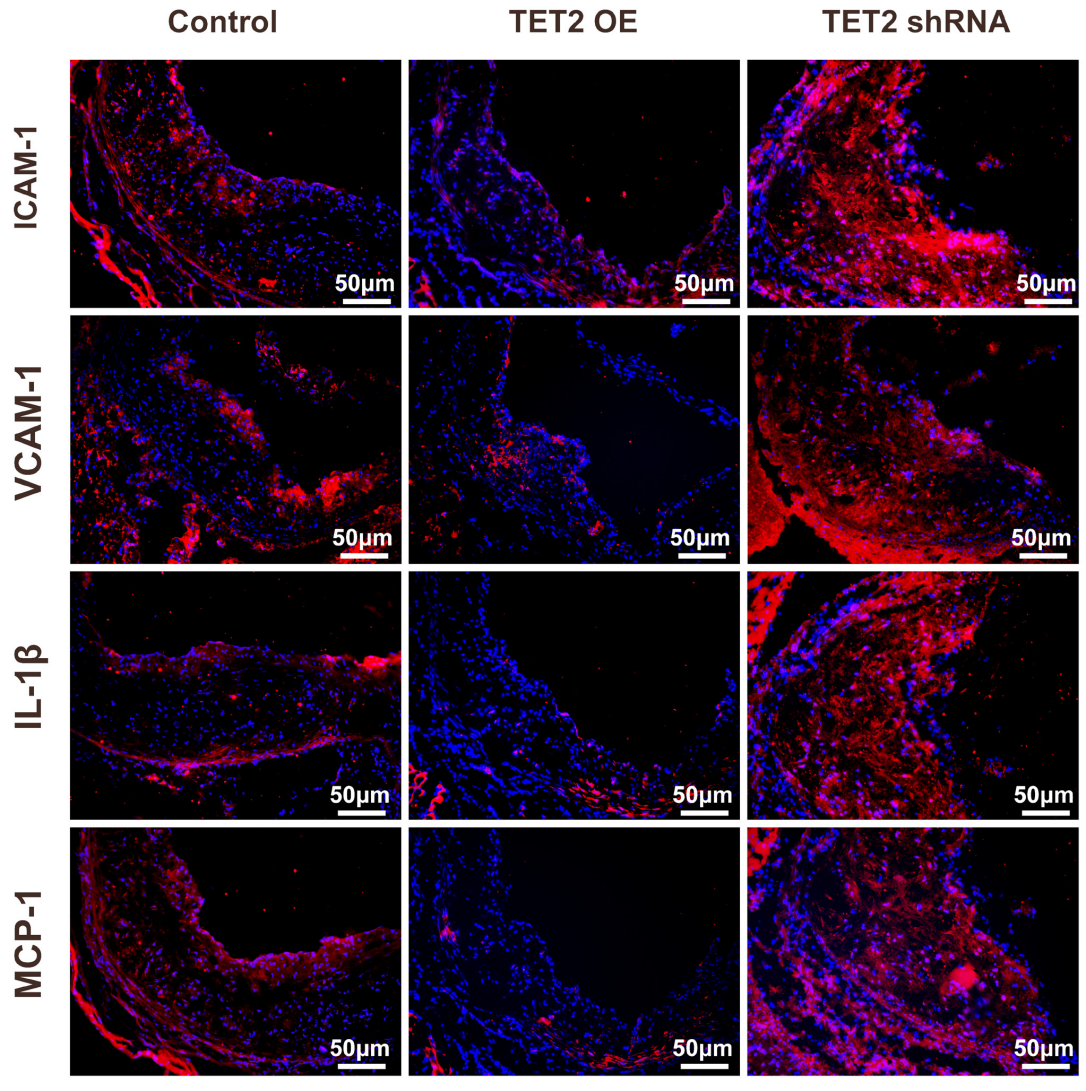
TET2 inhibited inflammation factor expression in the atherosclerotic lesions of high fat-fed ApoE^−/−^ mice Representative micrographs of immunofluorescence staining of ICAM-1, VCAM-1, IL-1β, and MCP-1 in atherosclerotic plaques were derived from the control, TET2 OE, and TET2 shRNA ApoE^−/−^ mice fed with a high-fat diet for 12 weeks (*n* = 10 each). Scale bar is 50 μM. TET2 OE:TET2 overexpression.

Atherosclerosis is a long-term chronic inflammatory process associated with inflammatory cells, adhesion molecules, inflammatory cytokines, and chemokines. Adhesion molecules, such as intercellular adhesion molecule 1 (ICAM-1) and vascular cell adhesion molecule 1 (VCAM-1), promote plaque instability, weaken the plaque fibrous cap, and accelerate plaque rupture and thrombosis. As shown in Figure 3, ICAM-1, VCAM-1, interleukine-1 beta (IL-1β) and monocyte chemotactic protein 1 (MCP-1) were abundantly expressed in atherosclerotic lesions as previously reported [16, 17]. The TET2 overexpression group showed significantly less positive staining areas for ICAM-1, VCAM-1, IL-1β, and MCP-1 than the control group. In addition, the TET2 shRNA group had a significant increase in the expression of these inflammation factors.

### Expression of TET2 is involved in the regulation of vascular cell autophagy in ApoE^−/−^ mice

Autophagy, a compensatory mechanism to maintain cell homeostasis, has recently been implicated as a protective mechanism during atherosclerosis [18]^.^ In the present study, the autophagy markers Beclin 1, LC3, and p62 were examined in TET2-overexpressing and TET2 shRNA-treated ApoE^−/−^ mice.

**Figure 4:**
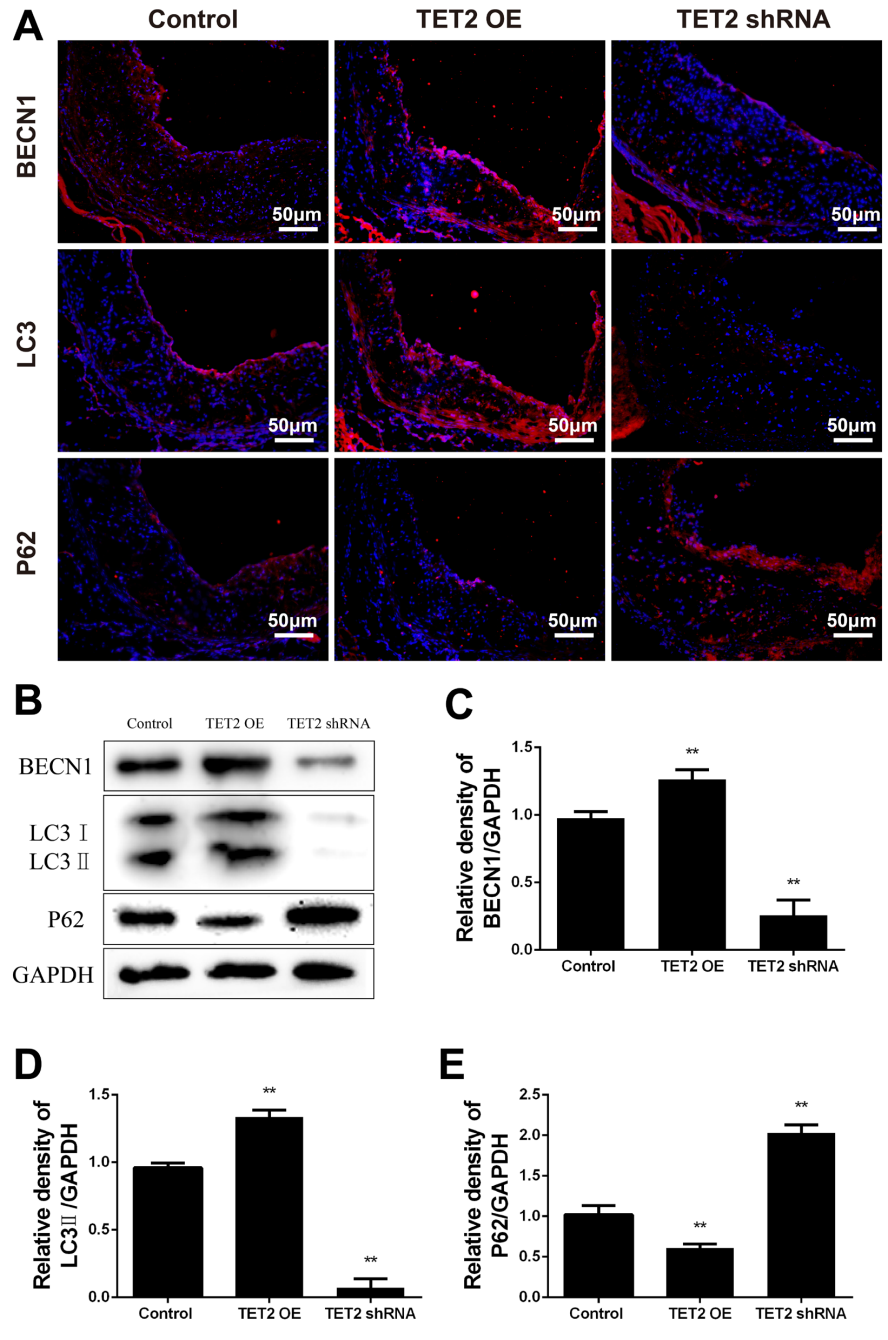
TET2 improved autophagy in the atherosclerotic lesions of high-fat-fed ApoE^−/−^ mice **A.** Representative micrographs of immunofluorescence staining of autophagy markers, such as Beclin 1, LC3, and p62, in atherosclerotic plaques were derived from the control, TET2 OE, and TET2 shRNA ApoE^−/−^ mice fed with high-fat diets for 12 weeks. DAPI was used for nuclear stains (blue). Scale bar is 50 μM. **B.** Representative Western blots showing the levels of Beclin 1, LC3, and p62 in aortic arch in the different groups. **C.**-**E.** Quantitative data showing the levels of autophagy-related proteins in aortic arch in the different groups. All results are expressed as mean ± S.D. (*n* = 3 each). *P < 0.05, **P < 0.01 *versus* control group. TET2 OE:TET2 overexpression.

Beclin 1 is a key regulator of autophagosome formation during autophagy. p62 is a ubiquitin-binding protein that interacts with LC3 to mediate the degradation of polyubiquitinated protein aggregates and mitochondria *via* the autophagy-lysosome pathway. The accumulation of p62 indicates defective autophagy lysosomes. The plaques and aortic archs in the TET2-overexpressing ApoE^−/−^ mice were characterized by an increased expression of Beclin 1 and LC3 and a decreased accumulation of p62 as compared with those in the control ApoE ^−/−^ mice (Figure 4). Consistent with these results, the expression of Beclin 1 and LC3 decreased and the accumulation of p62 increased in the TET2 shRNA-treated ApoE^−/−^ mice (Figure 4). These data suggest that TET2 is involved in vascular cell autophagy and that the loss of TET2 contributes to autophagy impairment during the progression of atherosclerosis.

### TET2 increases 5hmC and decreases 5mC in ox-LDL-treated human umbilical vascular endothelial cell lines (HUVECs)

**Figure 5:**
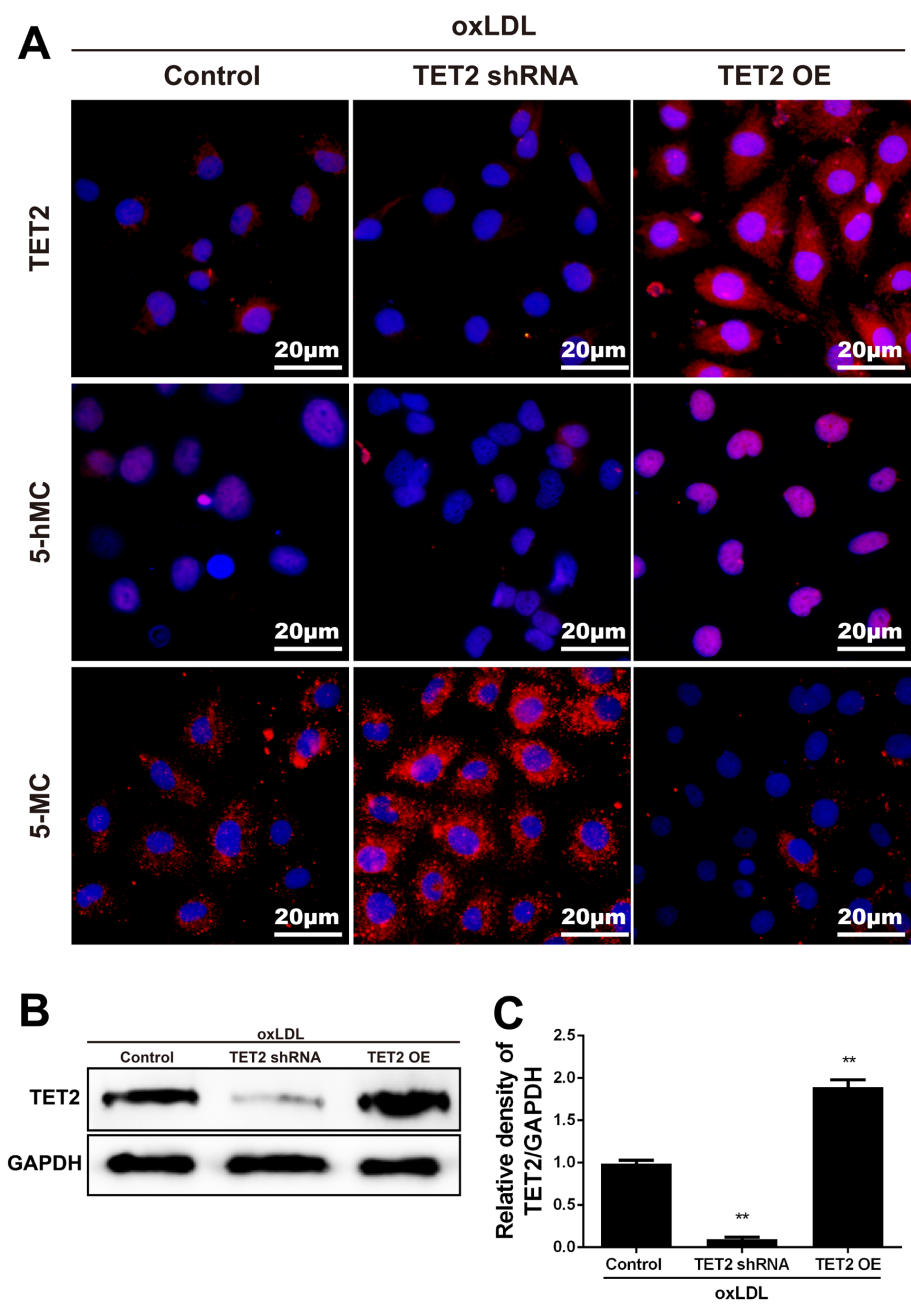
TET2 increased 5hmC level and decreased 5mC level in ox-LDL-treated HUVECs **A.** Representative immunostaining micrographs show that TET2 OE enhanced 5hmC level and reduced 5mC level in HUVECs treated with 50 mg/L ox-LDL for 24 h. Consistently, treatment with TET2 shRNA reduced 5hmC generation and increased 5mC generation. DAPI was used for nuclear staining (blue). Scale bar is 20 μM. **B.**-**C.** TET2 levels were determined in HUVECs with different treatments using Western blot. Values are mean ± SD from three independent experiments, each performed in triplicate. **P < 0.01 *versus* control group. TET2 OE:TET2 overexpression.TET2 OE:TET2 overexpression.

Ox-LDL-induced endothelium dysfunction initiates atherosclerotic lesion formation and progression. TET2 overexpression significantly increased 5hmC level but significantly decreased 5mC level in ox-LDL-treated HUVECs. This finding was further confirmed when the increased level of 5hmC and the decreased level of 5mC were inhibited by treatment with TET2 shRNA (Figure 5).

### TET2 improves autophagy and autophagic flux in ox-LDL-treated HUVECs

**Figure 6:**
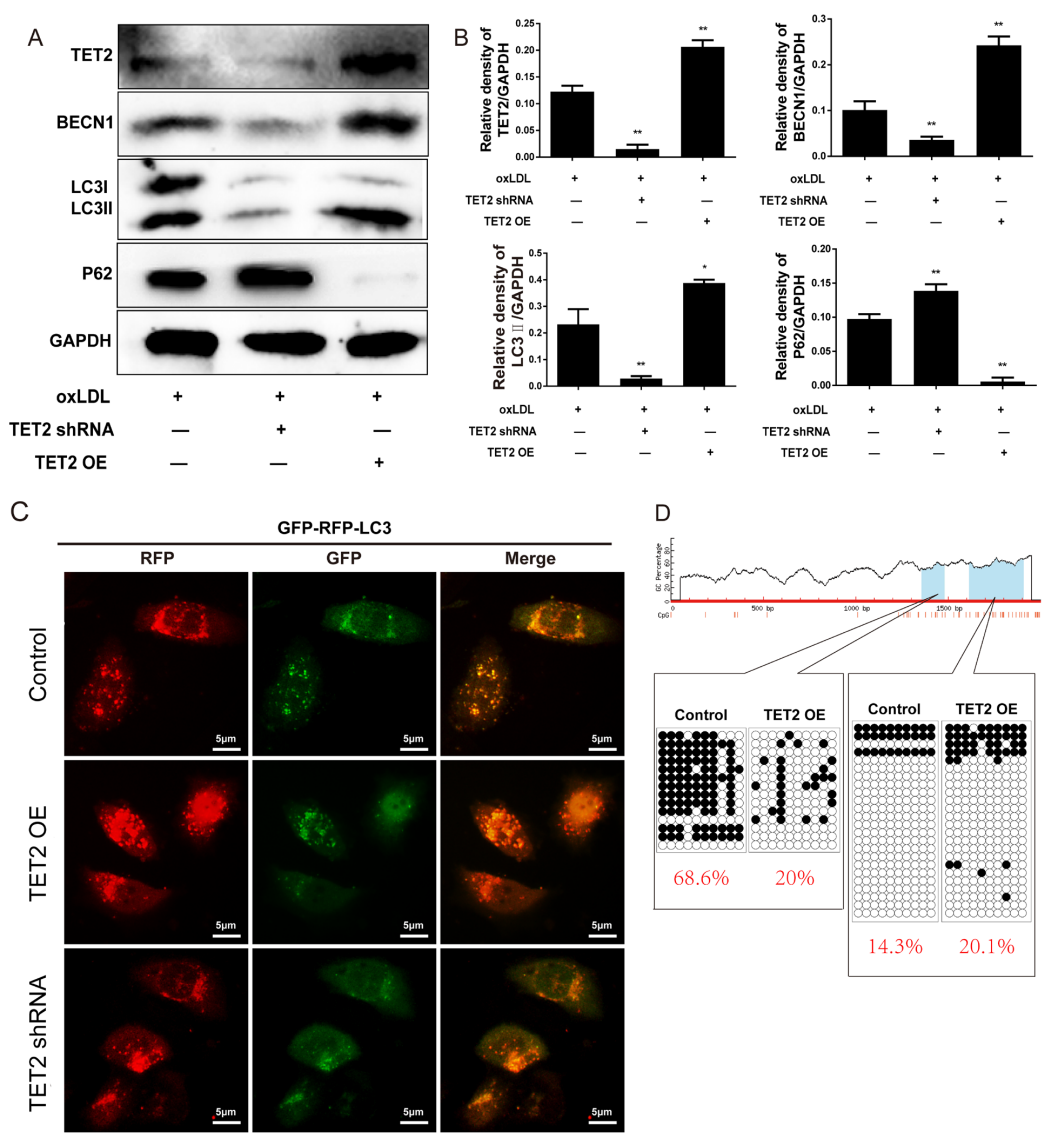
TET2 improved autophagy and autophagic flux in ox-LDL-treated HUVECs **A.** Representative Western blots showing the levels of Beclin 1, LC3 and p62 in HUVECs with different treatments. **B.** Quantitative data showing the levels of autophagy-related proteins in HUVECs with different treatments. All results are expressed as mean ± SD from three independent experiments, each performed in triplicate. *P < 0.05, **P < 0.01 *versus* ox-LDL-treated group. **C.** Representative images showing LC3 localization in HUVECs from different groups infected with the GFP-RFP-LC3 adenovirus for 24 h. Scale bar is 5 μM. **D.** Bisulfite sequence analysis was performed to compare the DNA methylation at the Beclin 1 promoter.

Oxidized lipoproteins lead to endothelial cell dysfunction by altering autophagy [19]. To determine the potential involvement of autophagy and autophagic flux in the effects of TET2 on ox-LDL-treated endothelial cells, we pretreated HUVECs with either TET2 overexpression or TET2 shRNA, followed by treatment with 50 mg/L ox-LDL for 24 h. As shown in Figures 6A and 6B, TET2 overexpression increased the levels of Beclin 1 and LC3II protein, but treatment with TET2 shRNA elicited the opposite effect. Meanwhile, p62 expression was decreased by TET2 overexpression and increased by treatment with TET2 shRNA, suggesting that TET2 regulates the autophagy of endothelial cells in the presence of ox-LDL treatment. The protective effect of autophagy requires autophagic flux through lysosomes [20]. Therefore, we used the tandem GFP-RFP-LC3 adenovirus to confirm the role of TET2 in the autophagy flux of endothelial cells. As shown in Figure 5C, TET2 overexpression resulted in more red puncta and less green puncta in endothelial cells compared with control cells, indicating that TET2 induced autolysosome formation. These results suggest that TET2 promotes autophagy flux in ox-LDL-treated HUVECs.

To elucidate the potential mechanisms underlying the effects of TET2 on autophagy in endothelial cells, we analyzed the methylation of the Beclin 1 promoter. Bioinformatic analysis showed two CpG islands (662-774 bp and 829-1225 bp) containing 14 and 21 CpG sites in the Beclin 1 promoter, respectively. Sequence analysis revealed that TET2 overexpression significantly decreased the average methylation rate of the first CpG island but not that of the second one (Figure 6D).

### TET2 inhibits inflammatory factor expression in ox-LDL-treated HUVECs

**Figure 7:**
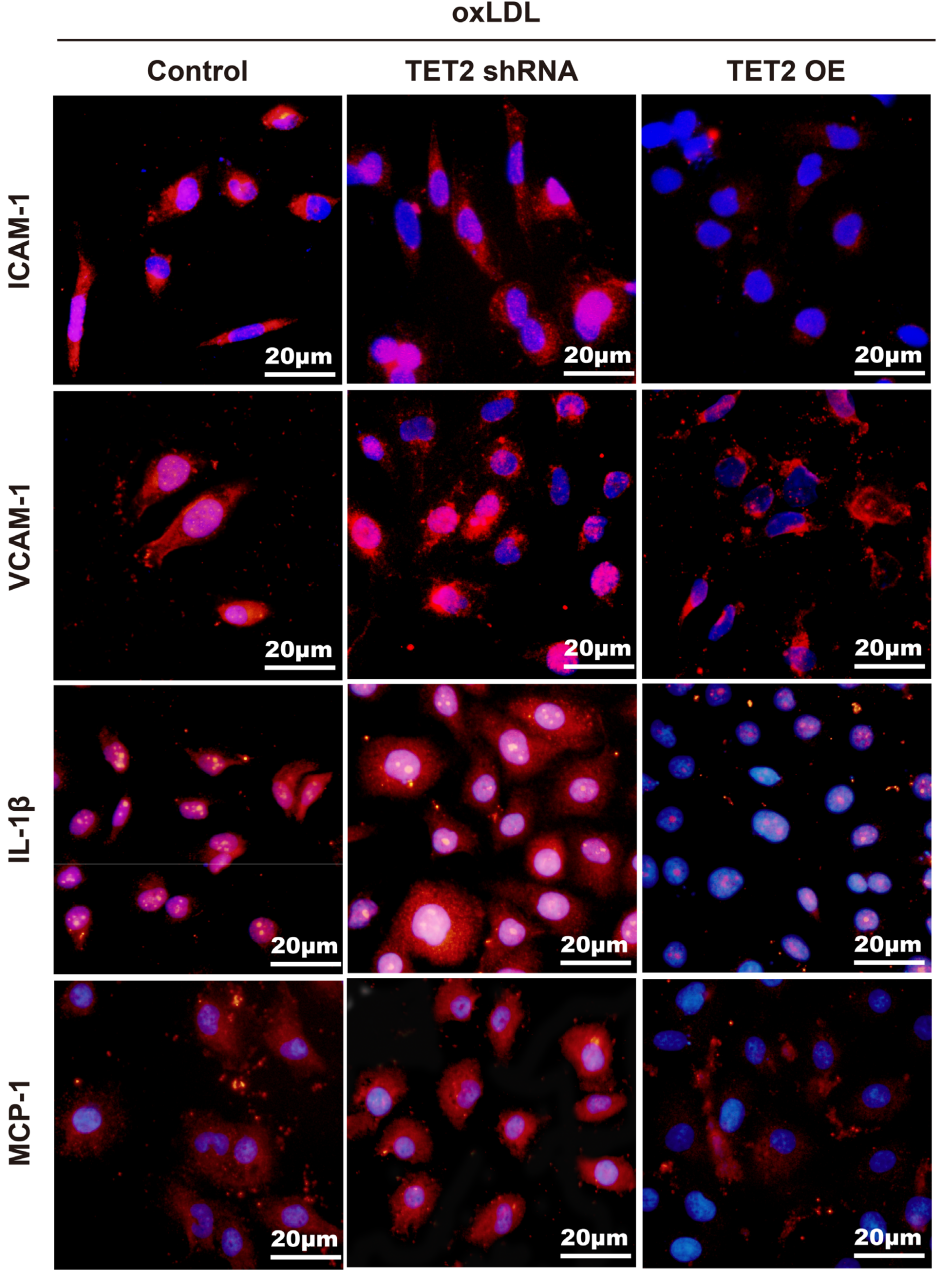
TET2 inhibited the expression of inflammatory factors in ox-LDL-treated HUVECs The increased levels of VCAM-1, ICAM-1, IL-1β, and MCP-1 in response to ox-LDL treatment were reduced by TET2 OE and further elevated by TET2 shRNA. Scale bar is 20 μM.

Atherosclerosis is characterized by impaired autophagy, and autophagy deficiency promotes atherosclerosis in part by activating the inflammasome [21]. We assessed the effects of TET2 on the expression of inflammatory factors in ox-LDL-induced HUVECs. The expression of ICAM-1, VCAM-1, IL-1β, and MCP-1 was significantly attenuated in response to TET2 overexpression and further increased by TET2 shRNA in ox-LDL-treated HUVECs (Figure 7).

## DISCUSSION

In this study, TET2 and its production decreased in atherosclerotic lesions. The overexpression of TET2 significantly downregulated the expression of inflammatory factors and the accumulation of macrophages in atherosclerotic lesions. This phenomenon resulted in the significant reduction of atherosclerosis. TET2 regulated autophagy and autophagic flux by modifying Beclin 1 promoter methylation in vascular endothelial cells. These data indicate that TET2 plays a unique role in the pathophysiological process of atherosclerosis.

TET2 participates in DNA methylation regulation by mediating oxidation in the conversion of 5mC to 5hmC, which epigenetically regulates gene expression by altering methylation-driven gene silencing. TET2^−/−^ mice show a dramatic reduction in 5hmC level and a concomitant increase in 5mC level in the genomic DNA of bone marrow cells [22]. The loss of TET2 and its production of 5hmC are closely associated with diverse types of cancers, such as myeloid leukemia, melanoma, and colorectal, breast, liver, and lung cancers. In the present study, the expression levels of TET2 and 5hmC obviously decreased in atherosclerotic lesions. Furthermore, TET2 contributed to the promoter methylation status of Beclin 1, a key regulator of autophagy that governs the autophagic process by regulating the generation of phosphatidylinositol3-phosphate and the subsequent recruitment of additional Atg proteins that orchestrate autophagosome formation [23]. Consistent with this finding, autophagy and autophagic flux in ox-LDL-treated endothelial cells were improved by TET2 overexpression and further impaired by TET2 shRNA. The vascular cell autophagy was also upregulated in ApoE−/− mice with TET2 overexpression and was decreased in ApoE−/− mice treated with TET2 shRNA. Overall, these observations suggest that TET2 is an important regulator in autophagy and autophagic flux in endothelial cells.

Inflammatory processes play a crucial role in all stages of atherosclerosis. In the early stage of atherosclerosis, entrapped ox-LDL in the vessel wall activates arterial endothelial cells and also recruits monocytes and T cells. Upon recruitment, monocytes can differentiate into macrophages. Autophagy acts as a negative regulator of inflammation. ATG16L1^−/−^ mice show high IL-1*β*and IL-18 levels in sera in response to LPS stimulation or during colitis [24]. The detailed molecular mechanisms by which autophagy regulates inflammation are not yet fully understood. In general, autophagy protects cells from excessive long-lasting inflammation in an indirect manner by allowing efficient clearance of damaged organelles (e.g., mitochondria,) and/or in a direct manner by suppressing proinflammatory complexes (e.g., mitochondrial ROS and DNA) [25, 26]. Autophagy impairment, which occurs in atherosclerotic lesions, promotes atherosclerosis at least partially by upregulating inflammation [27]. TET2 overexpression decreased the expression of inflammatory mediators, such as VCAM-1, ICAM-1, IL-1β, and MCP-1, in cultured endothelial cells. VCAM-1 and ICAM-1 are the major cytokines in monocyte adhesion that triggers the transformation from monocytes to macrophages within the vascular wall. MCP-1 is related to leukocyte recruitment, and IL-1β contributes to monocyte/macrophage differentiation. Consistently, the accumulation of macrophages within the lesions in HFD-fed ApoE−/− mice was significantly attenuated in response to TET2 overexpression. This role of TET2 was further confirmed by TET2 shRNA. The increased accumulation of macrophages is associated with an increased incidence of plaque rupture with subsequent thromboembolism, leading to ischemic neurological and cardiovascular events. Therefore, the loss of TET2 might result in vulnerable plaque formation, and TET2 upregulation may be beneficial to prevent atherosclerosis.

Ichiyama et al. [28, 29] showed that TET2 upregulates the expression of IL-10 and IL-17 by promoting DNA demethylation and repressing IL-6 expression through the recruitment of Hdac2 to IL-6 promoter. Therefore, TET2 might protect cells from inflammation by improving autophagy and direct anti-inflammation effect, suggesting that TET2 overexpression is an attractive strategy to treat atherosclerosis. Other experiments are required to elucidate the exact molecular mechanisms underlying TET2 effects and its roles in signal transduction pathways, which will be clinically targeted for the prevention or treatment of atherosclerosis.

## MATERIALS AND METHODS

### Animals

ApoE−/^−^ mice (male, 8 weeks old) were purchased from the Department of Laboratory Animal Science, Peking University Health Science Center, China. All mice were housed in a specific pathogen-free environment throughout the experiment and fed HFD (20% fat and 0.15% cholesterol; Qingzilan, Jiangsu, China) for 12 weeks. Mice were weighed before and after the HFD feeding. All animals in this study were housed in accordance with the guidelines of the Institutional Animal Care and Use Committee of the University of South China. All experimental procedures were approved by the Department of Medical Ethics at the University of South China.

### Lentivirus transduction

The lentiviruses containing shRNAs (sense-loop-antisense) for mouse TET2 or the TET2-expression vectors were purchased from GeneChem Corporation (Shanghai, China). Infection of ApoE−/− mice by lentivirus was performed by intravenous tail vein injection.

### Quantification of atherosclerosis lesions

Mouse hearts and whole aortas were removed and embedded in OCT, and 8 μm serial sections of the aortic root were cut for analysis of lesion morphologies with Oil Red O staining, Masson trichrome staining. The extent of the atherosclerotic lesions of each animal was calculated as the mean lesion area of five sections. Histological sections were quantified by the computer-assisted morphometric analysis using ImageJ software (National Institutes of Health, Bethesda, MD). Data were expressed as lesion size ± SEM.

### Plasma lipid analysis

Mice were fasted for 6 h prior to sacrifice. Blood samples were drawn by cardiac puncture using a 25G needle mounted on a 1 mL syringe and then collected in an EDTA-containing tube. Plasma was separated by centrifugation at 2000×*g* for 10 min and then stored at 4 °C. Plasma lipid concentrations, including total cholesterol (TC), high-density lipoprotein cholesterol (HDL), low-density lipoprotein cholesterol (LDL), and triglycerides (TG), were determined using an autoanalyzer (Cobas 6000; Roche, Nakakojo, Japan) in accordance with the manufacturer's procedures.

### Immunofluorescence staining

Immunofluorescence staining was performed as previously described [14]. In brief, after treatment with 3% H_2_O_2_ for 15 min and 10% normal goat serum for 30 min, sections or cells were incubated overnight at 4 °C with the following primary antibodies: TET2 (1:50, Santa Cruz Biotechnology), 5hmC (1:50, EPIGENTEK), 5mC (1:50, EPIGENTEK), α-smooth muscle actin (1:100, ProteinTech), Beclin 1 (1:100, bioworld), LC3 (1:100, ProteinTech), p62 (1:50, Santa Cruz Biotechnology), VCAM-1 (1:50, Santa Cruz Biotechnology), ICAM-1 (1:50, Santa Cruz Biotechnology), MCP-1 (1:200, Santa Cruz Biotechnology), and IL-1β (1:200, Santa Cruz Biotechnology). Sections or cells were then incubated with cy3-conjugated secondary antibody for 1 h at room temperature. Thereafter, the samples were counterstained with 4′,6-diamidino-2-phenylindol (DAPI) for the nuclei, followed by recording with a fluorescence microscope (IX70; Olympus, Tokyo).

### TET2 overexpression or knockdown in endothelial cells

HUVECs were seeded at 4×10^5^ cells/well in a six-well plate and then transfected with 5 μg of TET2 overexpression plasmid or TET2 shRNA plasmid (OriGene Technologies Inc.) using Lipofectamine LTX (Invitrogen) in accordance with the manufacturer's instruction. After 1 h, the transfection mixture was replaced with fresh growth medium. Any subsequent experiment on transfected cells was conducted 24 h after transfection.

### mRFP-GFP-LC3 adenovirus infection

mRFP-GFP-LC3 adenoviral vectors were obtained from HanBio Technology Co. Ltd. (HanBio, Shanghai, China). After reaching 50%-70% confluence in 24-well plates, cells were infected with mRFP-GFP-LC3 adenovirus in accordance with the manufacturer's instructions. In brief, HUVECs were incubated with growth medium supplied with the adenoviruses for 2 h at 37 °C and then incubated with a new medium for another 24 h at 37 °C. Autophagic flux was recorded with a fluorescence microscope (IX70; Olympus, Tokyo).

### Validation of differentially methylated genomic regions using bisulfite sequencing

Two differential CpG-rich regions of the Beclin 1 promoter were selected for bisulfite sequence. In brief, 1 μg of genomic DNA was bisulfite-converted using the EZ-96 DNA Methylation Kit (Zymo Research) in accordance with the manufacturer's protocol. Primers (5′-GTTTAGGTTGGAGTGTAGTGGTATG-3′, 5′-AACCACTACACTCCAACCTAAAC-3′, length 325; 5′-GAGTAGTTGGGATTAAGTTGGGATT-3′ 5′-ACTCCTAATCCACAAACTCACAAAC-3′, length 343) were designed using MethPrimer (http://www.urogene.org/cgi-bin/methprimer/methprimer.cgi). The PCR products were purified using SK8141 PCR Purification (Qiagen), cloned to pUC18-T Vector Systems, and then transformed to SK9307 cells. Independent white colonies (10-20) were selected and sequenced in GenomeLab™ GeXP Genetic Analysis System (Beckman Coulter). The bisulfite sequencing DNA methylation analysis software was used to analyze the sequencing data. The average of methylation percentage at each CpG site in each experimental group was used for analysis.

### Western blotting analysis

Cells were lysed, and protein was qualified using the BCA method as previously described [15]. Equal amounts (100 μg) of total protein were subjected to 10% sodium dodecyl sulfide-polyacrylamide gel electrophoresis and then transferred onto polyvinylidene difluoride membranes (Millipore, Bedford, MA). The membranes were incubated overnight at 4 °C with anti-TET2 (1:200 diluted), anti-Beclin 1 (1:1000 diluted), anti-LC3 (1:1000 diluted), anti-p62 (1:200 dilution), anti-VCAM-1(1:1000 dilution), anti-ICAM-1 (1:200 dilution), anti-MCP-1 (1:200 dilution), anti-IL-1β (1:200 dilution), or anti-GAPDH (1:1000 dilution) antibody. Then, the corresponding secondary antibody (peroxidase-conjugated anti-mouse or antigoat) was applied for 2 h. Enhanced chemiluminescence reagents (Perkin Elmer, Waltham, MA, USA) were used to detect the targeted antigen. The abundance of the targeted protein was analyzed using Labwork image analysis software. All experiments were performed at least three times.

### Statistical analysis

Data were presented as mean ± SD. The unpaired Student's *t* test or ANOVA was used to evaluate the significance of the differences. The CpG methylation rates of the Beclin 1 gene promoter were analyzed by chi-square test. *P* < 0.05 was considered to indicate statistical significance.

## SUPPLEMENTARY MATERIALS FIGURES


