# Supplementary material for: Tet methylcytosine dioxygenase 2 inhibits atherosclerosis via upregulation of autophagy in ApoE−/− mice
Source: Oncotarget. 2016 Nov 4;7(47):76423–36. doi: 10.18632/oncotarget.13121 (PMC5363520; doi:10.18632/oncotarget.13121)
Supplement: Supplementary file 1 [file oncotarget-07-76423-s001.pdf]

Tet methylcytosine dioxygenase 2 inhibits atherosclerosis *via* upregulation of autophagy in ApoE$^{-/-}$ mice

Supplementary Material

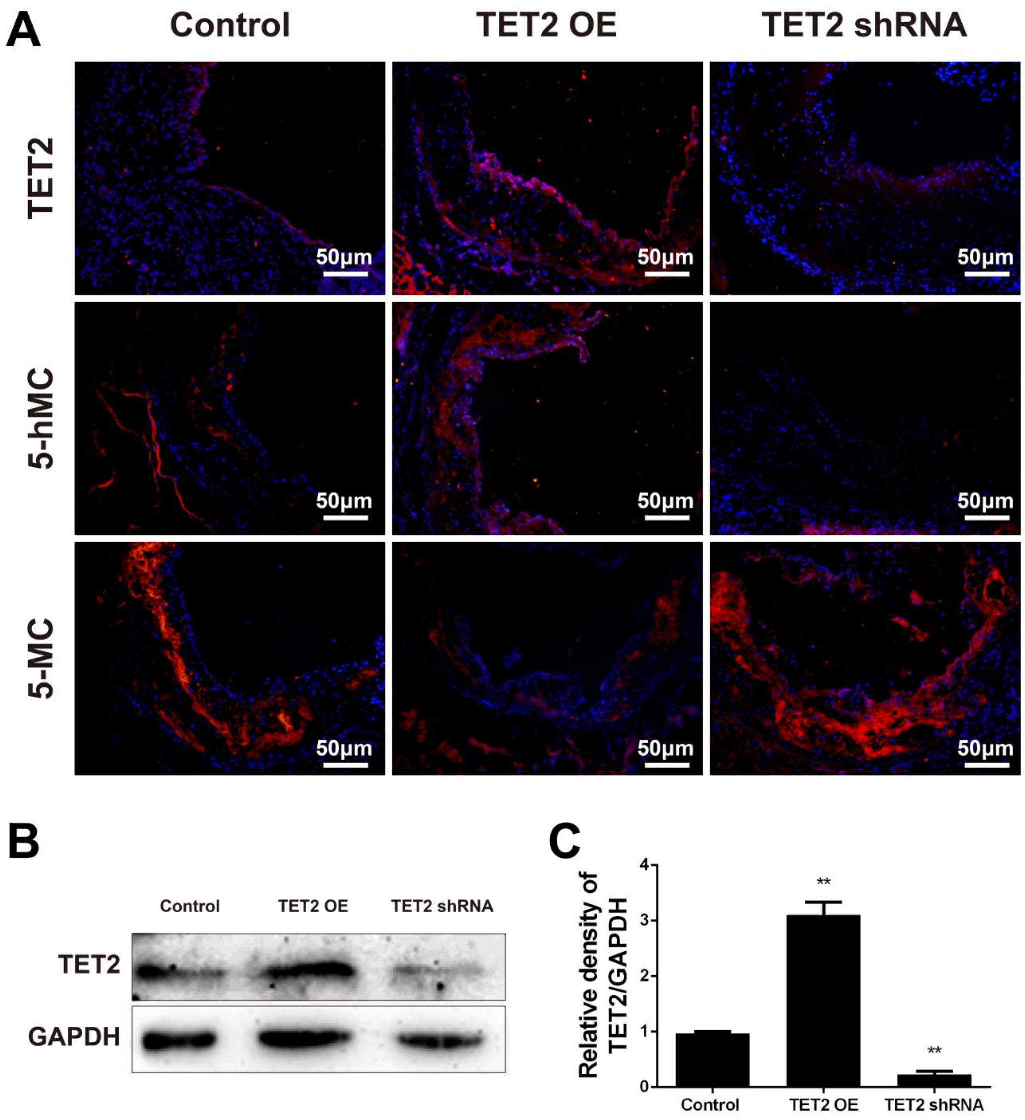

Supplementary Fig. 1. Expression of TET2, 5hmC, and 5mC in the atherosclerotic plaques in the control, TET2 OE, and TET2 shRNA-treated ApoE$^{-/-}$ mice. (A) Representative micrographs of immunofluorescence staining of TET2, 5hmC, and 5mC in atherosclerotic plaques were derived from the control, TET2 OE, and TET2 shRNA ApoE$^{-/-}$ mice fed with high-fat diet for 12 weeks. Scale bar is 50 μM. (B–C) TET2 levels were determined in aortic arch using Western blot. Values are mean ± SD. (n=3 each). $^{**}P<0.01$ versus control group. TET2 OE:TET2 overexpression.

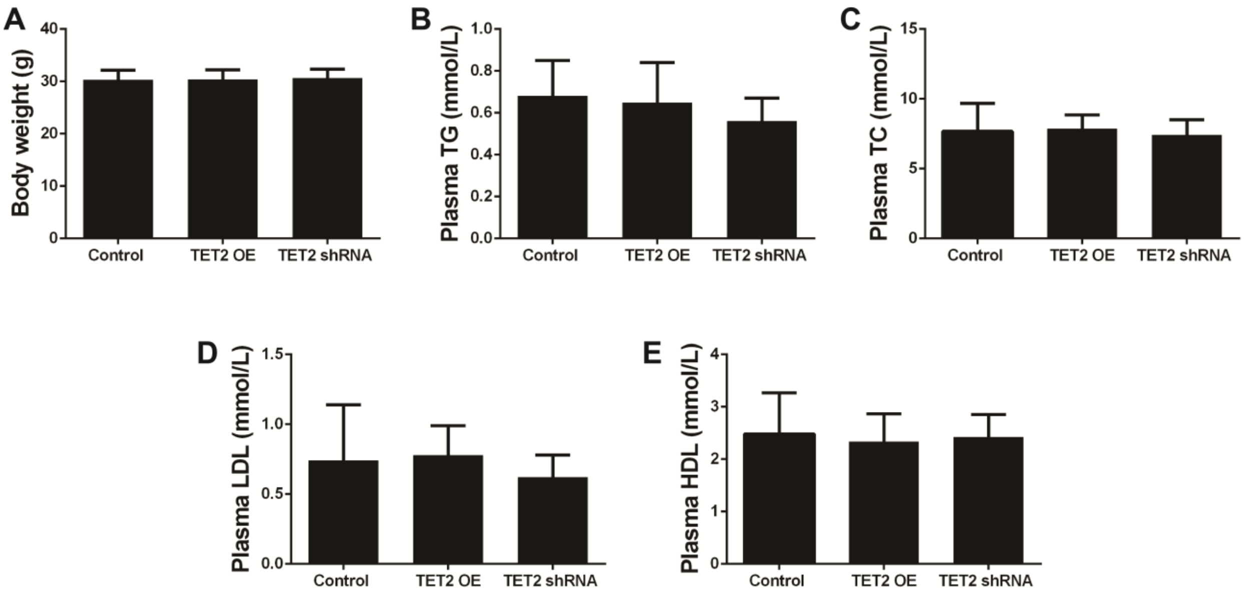

Supplementary Fig. 2. TET2 had no effect on body weight and plasma lipid profile in high-fat-fed ApoE−/− mice. Body weight and plasma lipid profile in ApoE$^{-/-}$ mice on a high-fat diet for 12 weeks. (A) Body weight ; (B) TG; (C) TC; (D) LDL; (E) HDL. Values are mean ± SD. (*n*=20 each).
